# Cost-effectiveness of out-of-hospital continuous positive airway pressure for acute respiratory failure: decision analytic modelling using data from a feasibility trial

**DOI:** 10.1186/s12873-021-00404-8

**Published:** 2021-01-25

**Authors:** Praveen Thokala, Gordon W. Fuller, Steve Goodacre, Samuel Keating, Esther Herbert, Gavin D. Perkins, Andy Rosser, Imogen Gunson, Joshua Miller, Matthew Ward, Mike Bradburn, Tim Harris, Maggie Marsh, Kate Ren, Cindy Cooper

**Affiliations:** 1 Health Economics and Decision Science, School of Health and Related Research, University of Sheffield, Regent Court, 30 Regent Street, Sheffield, S1 4DA UK; 2 Centre for Urgent and Emergency Care Research, School of Health and Related Research, University of Sheffield, Regent Court, 30 Regent Street, Sheffield, S1 4DA UK; 3 Clinical Trials and Research Unit, School of Health and Related Research, University of Sheffield, Regent Court, 30 Regent Street, Sheffield, S1 4DA UK; 4 Warwick Clinical Trials Unit, University of Warwick, Coventry, CV4 7AL UK; 5 West Midlands Ambulance Service, Trust Headquarters, Millennium Point, Waterfront Business Park, Waterfront Way, Brierley Hill, West Midlands DY5 1LX UK; 6 School of Medicine and Dentistry, Blizard Institute, Barts and The London School of Medicine and Dentistry, 4 Newark Street, London, E1 2AT UK; 7 Sheffield Emergency Care Forum, Clinical Research Office Sheffield, Royal Hallamshire Hospital, D Floor, Glossop Road, Sheffield, S10 2JF UK

**Keywords:** Cost-effectiveness, Continuous positive airway pressure, Acute respiratory failure

## Abstract

**Background:**

Standard prehospital management for Acute respiratory failure (ARF) involves controlled oxygen therapy. Continuous positive airway pressure (CPAP) is a potentially beneficial alternative treatment, however, it is uncertain whether this could improve outcomes and provide value for money. This study aimed to evaluate the cost-effectiveness of prehospital CPAP in ARF.

**Methods:**

A cost-utility economic evaluation was performed using a probabilistic decision tree model synthesising available evidence. The model consisted of a hypothetical cohort of patients in a representative ambulance service with undifferentiated ARF, receiving standard oxygen therapy or prehospital CPAP. Costs and quality adjusted life years (QALYs) were estimated using methods recommended by NICE.

**Results:**

In the base case analysis, using CPAP effectiveness estimates form the ACUTE trial, the mean expected costs of standard care and prehospital CPAP were £15,201 and £14,850 respectively and the corresponding mean expected QALYs were 1.190 and 1.128, respectively. The mean ICER estimated as standard oxygen therapy compared to prehospital CPAP was £5685 per QALY which indicated that standard oxygen therapy strategy was likely to be cost-effective at a threshold of £20,000 per QALY (67% probability). The scenario analysis, using effectiveness estimates from an updated meta-analysis, suggested that prehospital CPAP was more effective (mean incremental QALYs of 0.157), but also more expensive (mean incremental costs of £1522), than standard care. The mean ICER, estimated as prehospital CPAP compared to standard care, was £9712 per QALY. At the £20,000 per QALY prehospital CPAP was highly likely to be the most cost-effective strategy (94%).

**Conclusions:**

Cost-effectiveness of prehospital CPAP depends upon the estimate of effectiveness. When based on a small pragmatic feasibility trial, standard oxygen therapy is cost-effective. When based on meta-analysis of heterogeneous trials, CPAP is cost-effective. Value of information analyses support commissioning of a large pragmatic effectiveness trial, providing feasibility and plausibility conditions are met.

**Supplementary Information:**

The online version contains supplementary material available at 10.1186/s12873-021-00404-8.

## What is already known on this subject

- A recent meta-analysis suggested that prehospital continuous positive airway pressure (CPAP) delivered by emergency medical services is a potentially beneficial alternative to standard oxygen treatment for acute respiratory failure (ARF).
- Previous cost-effectiveness analysis suggest that prehospital CPAP could provide value for money. However, they were performed using ARF incidence estimates and clinical outcomes from the meta-analysis which may not be generalizable to the UK setting.

## What this study adds

- This study estimates the cost-effectiveness of prehospital CPAP in patients with ARF using data specific to the UK setting based on the findings of the ACUTE feasibility trial. Value of information analyses support commissioning of a large pragmatic effectiveness trial, providing feasibility and plausibility conditions are met.

## Background

Acute respiratory failure (ARF) is a common and life-threatening medical emergency [1]. ARF has substantial health services costs, with patients often requiring prolonged hospital stays, ventilatory support and critical care admissions. Incidence of ARF has been estimated at 80 cases per 100,000 per year; and ARF has substantial health services costs (estimated at £9.6 million per year in England [2]), with patients often requiring prolonged hospital stays [3], ventilatory support and critical care admissions [4, 5]^.^

Prehospital continuous positive airway pressure (CPAP) is a potentially beneficial alternative to standard oxygen treatment for ARF that could be delivered by emergency medical services [6]. A recent meta-analysis suggested that out-of-hospital CPAP could decrease mortality in ARF [2]. An economic evaluation using the estimates from the same meta-analysis suggested that while prehospital CPAP was more effective than standard care it was also more expensive [6]. Cost-effectiveness was consequently uncertain, with an incremental cost-effectiveness ratio of £20,514/quality adjusted life year (QALY) and a 49.5% probability of being cost-effective at the £20,000/QALY threshold. These estimates were predicated on the incidence of ARF and the accuracy of effectiveness data captured from the meta-analysis. However, included studies were at risk of bias and the methods used to deliver prehospital CPAP (physician or paramedics with online physician support) do not reflect systems that primarily use unsupported paramedics to deliver care, such as the UK National Health Service (NHS).

The Ambulance CPAP: Use, Treatment effect and economics (ACUTE) randomised controlled pilot trial [7, 8] was conducted to understand whether CPAP could be delivered effectively by unsupported paramedics and if it represents value for money. This study investigated the cost-effectiveness of prehospital CPAP compared with standard care for patients with ARF, using ARF incidence estimates and clinical outcomes from the ACUTE trial. Specific objectives were a) to estimate the cost-effectiveness of pre-hospital CPAP compared with standard care for patients with ARF, in terms of the costs and quality adjusted life years (QALYs) gained; b) Identify whether prehospital CPAP is likely to be cost-effective for patients with ARF at conventional willingness to pay thresholds; and c) evaluate the cost and value of undertaking further research by using value of information analyses.

## Methods

### Design

The decision problem was ‘which is the most cost-effective treatment strategy for patients presenting to UK ambulance services with ARF?’ A cost-utility economic evaluation was performed using a probabilistic decision analytic model to synthesise available evidence and compare alternative management strategies [9, 10]. Data from ACUTE trial is used in the base case – the rationale is that it is most relevant data for paramedic led services such as the NHS even though it is an imprecise effect estimate; and the aim of the model is to evaluate existing uncertainty around the decision problem relevant to this setting. Principles for economic evaluations outlined in the National Institute for Health and Care Excellence (NICE) Guide to the Methods of Technology Appraisal were followed [11]. The economic perspective was the UK health service in England and Wales with only direct treatment costs included. The model used 3.5% discount rate for costs and QALYs, and employed a lifetime horizon.

### Interventions

Any potentially relevant prehospital treatments that could feasibly be implemented in the UK health service for ARF were considered. However, due to the complexity of alternative forms of Non-Invasive Ventilation, only CPAP was judged as being a practicable alternative to standard oxygen practice. Interventions therefore comprised: pre-hospital CPAP provided by ambulance service clinicians and standard oxygen therapy i.e. without pre-hospital CPAP. Hospital management was assumed identical for both comparators.

### Model population and setting

The population consisted of a hypothetical cohort of patients with acute respiratory failure from any cause and potentially suitable for CPAP treatment and the setting was a representative ambulance service, such as West Midland Ambulance Service (WMAS) – setting for the ACUTE feasibility trial. Although this cohort could include patients with heterogeneous aetiology for ARF, including acute cardiogenic pulmonary oedema/heart failure, chronic obstructive pulmonary disease and pneumonia, for the purposes of modelling they were treated as a single group.

### Model structure

**Fig. 1:**
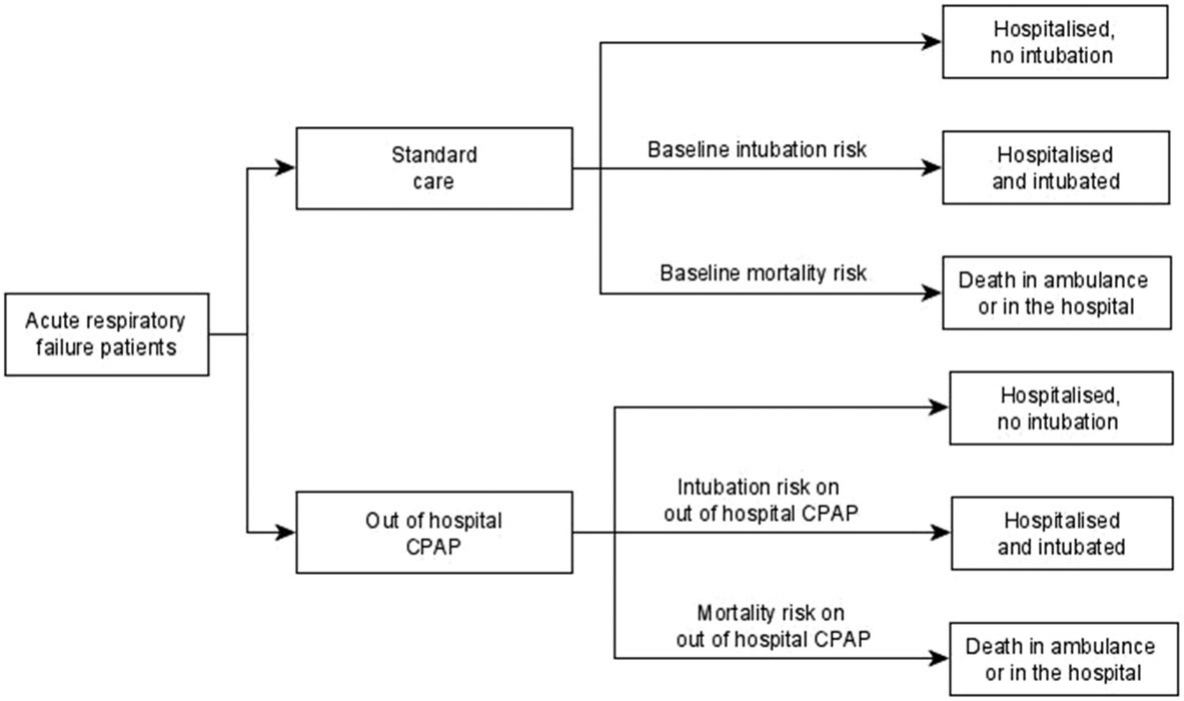
Structure of the decision analytic model

The model structure was based on a previously published economic model, as presented in Fig. 1. Patients received prehospital CPAP in the intervention group and standard care in the comparator group, and the treatment choice affected the probability of death and probability of intubation. The model assigned a baseline probability of intubation or death within 30 days for the standard care arm; and the intervention arm probabilities were estimated by applying log odds ratios (ORs) to the baseline risks. Patients who survived accrued lifetime QALYs and health care costs according to their life expectancy. Costs were also accrued through costs of intervention (i.e., out-of-hospital CPAP) and hospital treatment costs, which depended on whether the patient needed intubation. A summary of the parameters used in the model are reported in Table 1.

**Table 1:** Summary of model parameters

Parameter	Mean	Distribution or 95% CI	Source
**Baseline risks**			
Risk of mortality	0.257	Beta (9,26)	ACUTE
Risk of intubation	0.034	Beta (1,28)	ACUTE
**OR for prehospital CPAP**			
*Base case scenario (effectiveness parameters from ACUTE)*			
Log (Mortality OR)	0.145	Normal (0.145, 0.521)	ACUTE
Log (Intubation OR)	0.591	Normal (0.591, 1.403)	ACUTE
*Scenario using effectiveness parameters from NMA*			
Log (Mortality OR)	−0.916	Samples	ACUTE, HTA meta-analysis [2]
Log (Intubation OR)	−1.050	Samples	ACUTE, HTA meta-analysis [2]
**Life expectancy of patients**			
Lifetime years	2.67 years	Normal (2.67, 0.16)	3CPO trial [12], Clinical opinion
**Health related quality of life**			
Utility	0.6	Beta (640,425)	3CPO trial [12], Clinical opinion
**Costs (in £)**			
Prehospital CPAP	£33	Normal (£33, £3.3)	O-Two/SP [13], WMAS, Expert opinion
Hospitalisation	£3200	Gamma (80,40)	NHS Reference Costs [14]
Intubation	£3600	Gamma (90, 40)	HCHS index [15], Clinical opinion
Annual costs	£6000	Gamma (60, 100)	3CPO trial [12], HCHS index [15], Clinical opinion

*HCHS* Hospital and Community Health Services, *WMAS* West Midland Ambulance Service.

Analyses were performed using different effectiveness parameters. In the base case, effectiveness data from the ACUTE pilot trial were used as it is the most representative data for paramedic led services such as the NHS, even though it is an imprecise estimate. As a pragmatic trial, ACUTE used minimal exclusion criteria to select patients and CPAP was delivered using a simple disposable unit (O-Two [13]) by ambulance clinicians without extended or specialist training. A scenario analysis was undertaken using effectiveness parameters a recent network meta-analysis synthesising previously published experimental data, updated with results from the ACUTE study, using identical methods to those previously reported. The other studies included in the meta-analysis used less pragmatic inclusion criteria, more complex CPAP delivery systems and involved physicians or paramedics with online physician support to deliver the interventions. From the perspective of an ambulance service based on unsupported ambulance clinicians, the ACUTE data provide a relatively imprecise estimate of effectiveness (i.e. how the intervention works in usual practice), while the meta-analysis provides a more precise estimate of efficacy (i.e. how the intervention could work in certain circumstances).

### Data

Parameter estimates, distributional forms and data sources are summarized in Table 1. The baseline risk of mortality was modelled using the 30-day mortality data from control arm of the ACUTE pilot trial which reported 9 deaths (25.7%, *n* = 35, complete case, modified intention to treat analysis set). The intubation risk, which determines whether critical care admission is required, was also modelled using the data from control arm of the ACUTE trial, which reported one intubation (3.4%, *n* = 29, complete case, modified intention to treat analysis set).

The base case analysis used relative effectiveness results from the ACUTE pilot trial for mortality and intubation. The odds ratios (OR) for effectiveness of CPAP for reducing mortality and intubation were 1.2 (95% CI 0.4 to 3.2) and 1.8 (95% CI 0.2 to 40.1) respectively. A scenario analysis was also performed using network meta-analysis [2] revised with results from the ACUTE study, using identical methods to those previously reported. The odds ratios (OR) with 95% credible intervals for reducing mortality and intubation were 0.5 (95%CI 0.2 to 1.4) and 0.4 (0.1 to 0.9) respectively [8].

Lifetime QALYs for surviving patients were estimated by multiplying the life years with representative quality of life using same estimates as in the previous economic model [2]; both derived from the 3CPO trial [12]. Discounted life expectancy was estimated at 2.67 years and the mean utility value was 0.6.

The costs included in the model are for prehospital CPAP, intubation, hospitalization, and lifetime care for patients. We estimated the costs of prehospital CPAP at an ambulance service level and converted these into a cost per patient according to a 5-year depreciation period. These costs included those for initial equipment, implementation, and ongoing maintenance. This resulted in a final CPAP cost per patient ranging from £26.53 to £39.57, which was assumed to be normally distributed around the mean of £33.00 with a standard deviation of £3.30. More details about the prehospital CPAP costing is provided in Additional file 1.

The cost of intubation was estimated in the previous HTA economic model by multiplying intensive care unit costs by the average length of stay for intubation assumed to be 5 days. These costs were inflated and this resulted in a mean annual cost of £3600 which was parameterised as a gamma distribution with an alpha of 90 and a beta of 40, after consultation with ACUTE study clinical experts.

The hospitalisation costs were estimated as weighted average costs of non-intubated patients in the ACUTE trial that received NIV in hospital (approximately 42.5% between both arms) and that did not, which corresponded to patients with NHS Reference Cost codes DZ27S (respiratory Failure without Interventions, with CC Score 11+) and patients with code DZ27P (respiratory Failure with Single Intervention, with CC Score 11+), respectively based on expert clinical input. Thus, the mean inpatient admission cost for hospitalisations was calculated as weighted average of the costs of patients with DZ27S and DZ27P, from the NHS Reference Costs for 2016–17 [14], resulting in a mean cost of £3200 and represented as gamma distribution with an alpha of 80 and a beta of 40.

Lifetime costs of survivors were estimated using the annual costs and the discounted life expectancy of patients captured from the 3CPO trial [12], which were inflated resulting in a mean annual cost of £6000. In the model, this annual cost was parameterised as a gamma distribution with an alpha of 60 and a beta of 100, after discussions with ACUTE clinical experts. It was assumed that the lifetime costs were the same for all survivors, irrespective of whether they were in the standard care or prehospital CPAP arm.

### Cost-effectiveness analysis

The cost-effectiveness was estimated using both the incremental cost effectiveness ratios (ICER) and the net monetary benefit (NMB) approaches. The ICER is calculated as the mean incremental cost divided by the mean incremental benefits, computed by comparing to the next most effective alternative. The willingness to pay threshold (λ) is the amount of money that the decision-maker is willing to pay to gain an additional QALY [16]. The usual threshold for decision-making in the UK is based on information from NICE, and considered to be £20,000 per QALY as detailed in NICE HTA guidelines. This effectively means that NICE will recommend an intervention for funding if it can deliver health gain at a cost no greater than £20,000 per QALY compared to the next most effective alternative. The NMB framework transforms cost-effectiveness results to a linear scale; it is defined as the QALYs multiplied by a value for the QALYs (e.g. £20,000) minus the costs of obtaining them: NMB = (QALYs × λ) – cost. The strategy with the highest expected incremental net monetary benefit is the most cost-effective [9, 17].

### Probabilistic sensitivity analysis

In order to account for the uncertainty in model inputs a probabilistic sensitivity analysis (PSA) was conducted using Monte Carlo simulation [9, 17, 18] Multiple model runs were performed, each with a random draw from every parameter’s probabilistic distribution, thus evaluating the full range of cost-effectiveness results possible given the uncertainty on model inputs [9, 17, 18]. Mean ICERs calculated from the average expected costs and effects over all model runs, were computed and compared with cost-effectiveness thresholds to inform adoption decisions. The incremental costs and of each model run were depicted graphically on a cost-effectiveness plane. A cost-effectiveness acceptability curve (CEAC), plotting a relevant range of λ values against the probability that each intervention was the most cost-effective, was also graphed to summarise the uncertainty of PSA results [19].

### Value of information analysis

The population expected value of perfect information (EVPI) places an upper limit on what healthcare system should be willing to pay for additional evidence to remove decision uncertainty i.e. EVPI informs the future total value of addition research relating to a specific decision problem [9, 12]. The population expected value of partial perfect information (EVPPI) is the value in improving the precision of estimates of parameters, or groups of parameters.

Individual level expected value of information metrics were initially calculated for both the base case and updated meta-analysis scenario analysis. EVPI for individual patients was directly calculated directly from the model PSA output using standard formulas [9] and individual EVPPI values were estimated by using 2 level Monte Carlo simulation techniques [20]. Assumptions on ARF incidence (11,000 patients per year in England and Wales) and health technology lifespan (5 years) were used to compute population level results.

## Results

### Base case cost-effectiveness results: effectiveness estimates from ACUTE pilot trial

The base case analysis indicated that the prehospital CPAP strategy was cheaper and less effective than standard care. The ICER was therefore interpreted as the incremental costs and QALYs of standard care compared to CPAP (because the ICER is calculated by comparing to the next most effective alternative).

The mean expected costs of standard care and prehospital CPAP were £15,201 and £14,850 respectively. The corresponding mean expected QALYs were 1.190 and 1.128. The mean ICER, estimated as standard care compared to CPAP, was £5685 per QALY. Given the typical NICE threshold of £20,000 per QALY, the base case analysis indicates that standard care is cost-effective because it gains QALYs with an acceptable ICER compared to CPAP. Table 2 summarises mean expected costs and QALYS, ICERs and NMB for the base case analysis.

**Table 2:** Mean expected costs and QALYS, ICERs and NMB for base case and scenario analyses

Strategy	Mean Cost	Mean QALYs	Mean ICER	Mean NMB	Mean incremental NMB*	Probability most cost effective*
Base case: ACUTE pilot trial effectiveness data						
Standard Care	£15,201	1.190	£5685^a^	£8598	£883^a^	0.67
Prehospital CPAP	£14,850	1.128	–	£7715	–	0.33
Scenario analysis: Updated network meta-analysis effectiveness estimates						
Standard Care	£15,201	1.19	–	£8598	–	0.06
Prehospital CPAP	£16,722	1.35	£9712^b^	£10,209	£1612^b^	0.94

^a^Mean ICER/incremental NMB estimated as standard care compared to CPAP. *Assuming a threshold value of £20,000 per QALY. ^b^ Mean ICER/incremental NMB estimated as CPAP compared to standard care

**Fig. 2:**
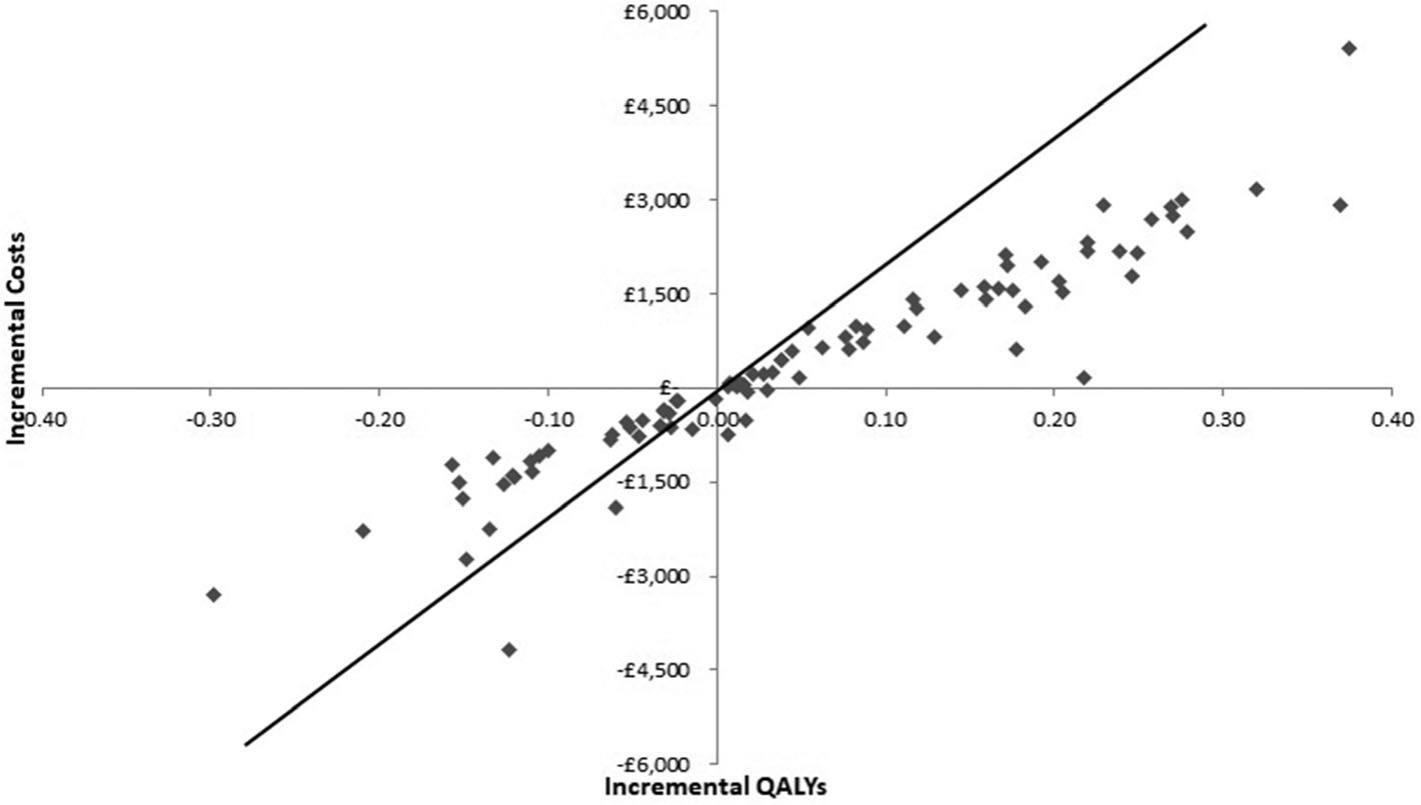
Cost-effectiveness plane showing incremental costs and QALYs for standard care compared to prehospital CPAP for base case analysis using ACUTE effectiveness data

**Fig. 3:**
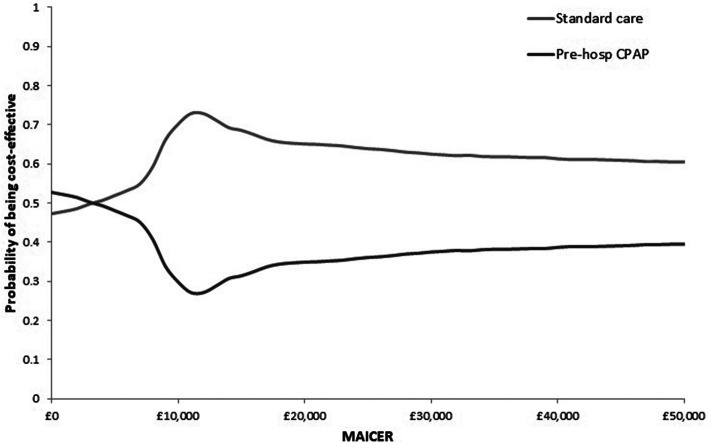
Cost-effectiveness acceptability curve for the base case analysis using ACUTE effectiveness data. *MAICER: maximum acceptable incremental cost-effectiveness ratio

Scatterplots of the incremental expected costs and QALYs from the PSA are shown in Fig. 2, which suggests a large degree of uncertainty, reflected in the dispersal of PSA simulations, falling in both the North East and South West quadrants of the cost-effectiveness plane. The base case cost-effectiveness acceptability curve (CEAC) is shown in Fig. 3. At thresholds less than £5000 per QALY, prehospital CPAP was the most cost-effective strategy in the majority of model runs, however, at thresholds beyond £5000 per QALY, standard care has more probability of being cost-effective and at the £20,000 per QALY threshold, standard care was most likely to be cost-effective (67%).

Scenario analyses results: effectiveness estimates from Updated network meta-analysis.

A scenario analysis indicated that the prehospital CPAP strategy was more expensive and more effective than standard care. The ICER was therefore interpreted as the incremental costs and QALYs of prehospital CPAP compared to standard care (because the ICER is calculated by comparing to the next most effective alternative).

The mean expected costs of standard oxygen therapy and prehospital CPAP were £15,201 and £16,722 respectively. The corresponding mean expected QALYs were 1.19 and 1.35. The mean ICER, estimated as prehospital CPAP compared to standard care, was £9712 per QALY. Given the typical NICE threshold of £20,000 per QALY, in this analysis it would be concluded that prehospital CPAP is cost-effective because it gains QALYs with an acceptable ICER compared to standard care. Table 2 summarises mean expected costs and QALYS, ICERs and NMB for this scenario analysis.

**Fig. 4:**
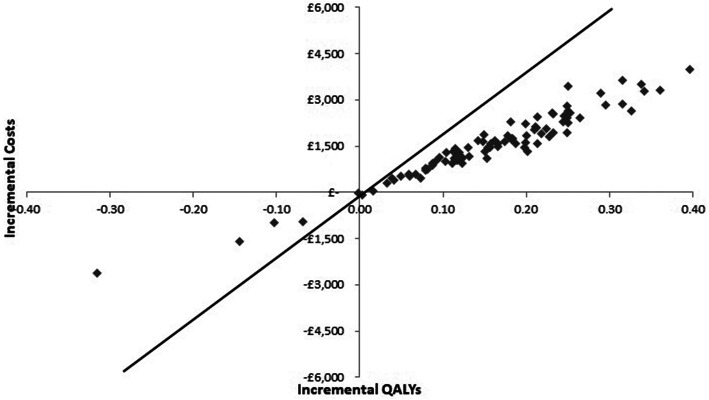
Cost-effectiveness plane showing incremental costs and QALYs for prehospital CPAP compared to standard care in the updated network meta-analysis scenario analysis

**Fig. 5:**
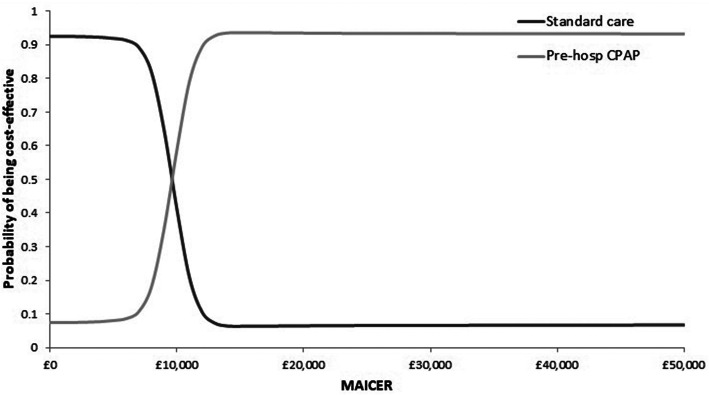
Cost-effectiveness acceptability curve for the updated meta-analysis scenario analysis. *MAICER: maximum acceptable incremental cost-effectiveness ratio

Scatterplot of the incremental expected costs and QALYs from the PSA are shown in Fig. 4, which indicates much less uncertainty than the base case, with incremental expected costs and effects clustering in the North East quadrant of the cost-effectiveness plane. The base case cost-effectiveness acceptability curve (CEAC) is shown in Fig. 5. The percentage of model runs in which prehospital CPAP was the most cost-effective strategy did not exceed 50% at thresholds less than £10,000/QALY, however, at the £20,000 per QALY prehospital CPAP was highly likely to be the most cost-effective strategy (94%).

Value of information analyses demonstrated there was considerable uncertainty about whether to adopt prehospital CPAP (see Additional file 2 for more details). In the base case analysis, the population EVPI indicated it would be worth spending up to £16.5 million on research investigating the effectiveness of prehospital CPAP in ARF. This is higher in comparison to a population EVPI of £3.72 million in the updated meta-analysis scenario analysis. EVPPI analyses indicated effectiveness of prehospital CPAP on mortality was the only important variable for future research, with population of EVPPI of £16.5 million and £3.72 million respectively in the base case and updated meta-analysis scenario analysis.

## Discussion

### Summary of findings

The base case analysis, using CPAP effectiveness estimates from the ACUTE pilot trial, indicated that standard oxygen therapy strategy was more effective (mean incremental QALYs of 0.062), but also more expensive (mean incremental costs of £351), than prehospital CPAP with a mean ICER, estimated as standard care compared to CPAP, of £5685 per QALY. A scenario analysis, using effectiveness estimates from an updated meta-analysis, suggested that prehospital CPAP was more effective (mean incremental QALYs of 0.157), but also more expensive (mean incremental costs of £1522), than standard care with a mean ICER, estimated as prehospital CPAP compared to standard care, of £9712 per QALY.

### Interpretation

The decision analytic model showed that the key determinant of cost-effectiveness is whether prehospital CPAP is effective or not in reducing mortality. This contrasts to the preceding economic model which suggested that the incidence of ARF was very important, secondary to its influence on prehospital CPAP costs. The O-Two CPAP device used in the ACUTE model is much cheaper and requires less training than the system previously examined [13], meaning that the costs of providing prehospital CPAP and thus the incidence of ARF is no longer critical in determining cost-effectiveness.

There is significant uncertainty around what is the most valid and applicable effectiveness estimate for prehospital CPAP. The base case analysis, using ACUTE pilot trial effectiveness data, suggested that prehospital CPAP was cheaper than standard care. This arises from increased short term mortality with fewer patients incurring critical care or lifetime health costs. However, this also results in fewer lifetime QALYs, and at the conventional £20,000 threshold there is a 67% probability that standard care is the most cost-effective option. The ACUTE pilot trial should be more representative of NHS ambulance services, but the low sample size gives very imprecise effectiveness estimates and leaves considerable uncertainty around cost-effectiveness, reflected in the large population EVPPI for the mortality effectiveness parameter. Whilst the scenario analysis using updated meta-analysis effectiveness data gives the opposite conclusion to the base case and suggests that CPAP is highly likely to be cost-effective at a threshold of £20,000 per QALY, the population EVPI and EVPPI for CPAP effectiveness still remains high suggesting that uncertainty in the effectiveness parameter.

Overall, the economic evaluation indicates that cost-effectiveness is principally dependent on the clinical effectiveness of CPAP and it is worthwhile for future research to reducing the uncertainty in this parameter, as suggested by the value of information analyses. In both the scenarios, the population EVPI and EVPPI for CPAP effectiveness remained high, supporting the commissioning of a large pragmatic effectiveness trial, providing feasibility and plausibility conditions are met.

### Generalisability of findings

The economic model follows recommendations from NICE and should have good external validity to UK settings. However, it may not be possible to generalise these results to other populations and jurisdictions with different health systems due to the potential differences in the effectiveness, costs, type of patients and service pathways. The ACUTE trial enrolled patients with non-differentiated ARF and used a specific disposable CPAP unit. Base case cost-effectiveness estimates could therefore differ if CPAP is used more selectively, or if alternative CPAP systems are used. Furthermore, the analyses were from a health care perspective using cost per QALY approach so these results may not be generalisable to settings using other perspectives (e.g. societal perspective) or other economic evaluation methods (e.g. cost-benefit analysis or cost-consequence analysis).

### Comparison to literature

Only one other economic evaluation of prehospital NIV for patients with ARF is available [21]. However meaningful comparison with the current study is difficult. In-hospital effectiveness data were used rather than prehospital data; outcomes were valued as lives saved rather than QALYs; the model only used a 1-year time horizon; and probabilistic sensitivity analysis was not performed.

### Limitations

This economic evaluation updated a previously published decision analytic model [2] and followed NICE base case recommendations, taking the perspective of the NHS in England and Wales, valued outcomes as QALYs, used a lifetime horizon, and included probabilistic sensitivity analysis [9, 17, 18]. The strengths included detailed costing at the level of the ambulance service; and use of relevant existing data sources to estimate key population, cost and outcome parameters. Decision uncertainty was explored in scenario analyses using different effectiveness estimates. Using ACUTE data, directly relevant to the study setting, for key ARF and effectiveness parameters in the base case, helped overcome the main limitations of the preceding economic analysis [2], which was reliant on potentially estimates from less pragmatic trials that used physicians and paramedics with online support to deliver the intervention. However, the low sample size in the ACUTE pilot trial resulted in very imprecise effectiveness estimates and leaves considerable uncertainty around cost-effectiveness. In order to address this uncertainty, scenario analysis was performed using the updated meta-analysis effectiveness data. In both analyses, there was large population EVPPI for the mortality effectiveness parameter suggesting substantial uncertainty in the effectiveness.

There are limitations in the model design and parameterisation which could challenge the internal validity of results. Within the modelled population there will be a considerable diversity of patients with differing characteristics, underlying diagnoses, and prognoses. Applying a cohort methodology, with consequent use of mean values, impeded an examination of uncertainty due to heterogeneity. However, competing management strategies are service level interventions, and hence would be applied to the entire population presenting with ARF and ostensibly eligible for CPAP. Exploration of heterogeneity, for example the cost-effectiveness in different underlying diseases, is therefore less relevant. The model assumed that the proportion of patients that would receive NIV in hospital was similar in both arms, irrespective of whether patient received prehospital CPAP. This appears plausible based from the limited ACUTE pilot trial data, but it is conceivable that there could be an association between the effectiveness of treatment during the EMS interval and ED management.

It was also assumed that the lifetime QALYs were same for all survivors, irrespective of whether they were in the standard care or prehospital CPAP arm. There was a limited evidence base available to parameterise lifetime QALYs and costs of care, with data provided by the 3CPO trial [12]. This study enrolled patients with pulmonary oedema receiving emergency department NIV, rather than the undifferentiated EMS ARF cases relevant for prehospital CPAP. However, baseline characteristics of participants in 3CPO appear similar to those included in ACUTE. Although unproven, this appears to be reasonable, as CPAP would only be expected to help with acute presentations and short term outcomes, rather than modify underlying chronic diseases.

## Conclusions

The cost-effectiveness of prehospital CPAP depends upon the estimate of effectiveness. When based on a small pragmatic feasibility trial, standard oxygen therapy is cost-effective. When based on meta-analysis of heterogeneous trials, CPAP is cost-effective. Value of information analyses support the commissioning of a large pragmatic effectiveness trial, providing feasibility and plausibility conditions are met.

## Supplementary Information

**Additional file 1: Appendix 1.** Estimating the costs of prehospital CPAP. Provides more details about the prehospital CPAP costing.

**Additional file 2: Appendix 2.** Value of Information analyses. Provides more detail about the methods and results of value of information analyses.

**Additional file 3: Supplementary file** - CHEERS Checklist.docx. Consolidated Health Economic Evaluation Reporting Standards (CHEERS) Checklist. Presents the completed CHEERS checklist for reporting health economic studies.

## Data Availability

Requests for data and code should be made to the corresponding author. Although specific consent for data sharing was not obtained the authorship group will consider will release of data on a case by case basis following published guidelines.

## Abbreviations

ACUTE: Ambulance CPAP: Use, Treatment effect and economics
ARF: Acute respiratory failure ()
CEAC: Cost-effectiveness acceptability curve
CPAP: Continuous positive airway pressure
EVPI: Expected value of perfect information
EVPPI: Expected value of partial perfect information
HCHS: Hospital and Community Health Services
ICER: Incremental cost effectiveness ratio
NICE: National Institute for Health and Care Excellence
NHS: National Health Service
NMB: Net monetary benefit
OR: Odds ratios
PSA: Probabilistic sensitivity analysis
QALY: Quality adjusted life year
WMAS: West Midland Ambulance Service
