# Supplementary material for: Cost-effectiveness of out-of-hospital continuous positive airway pressure for acute respiratory failure: decision analytic modelling using data from a feasibility trial
Source: BMC Emerg Med. 2021 Jan 25;21:13. doi: 10.1186/s12873-021-00404-8 (PMC7836588; doi:10.1186/s12873-021-00404-8)
Supplement: Supplementary file 1 — Additional file 1: Appendix 1. Estimating the costs of prehospital CPAP. Provides more details about the prehospital CPAP costing. [file 12873_2021_404_MOESM1_ESM.docx]

## Appendix 1: Estimating the costs of prehospital CPAP

There is a paucity of data on the total costs of providing prehospital CPAP and thus, bottom up costing methods were used, updating the previous economic model values with more relevant and contemporary data. There are a number of costs involved in providing prehospital CPAP, comprising initial and ongoing training and equipment costs. These total costs were converted into a cost per patient based on a five-year depreciation period (i.e. assuming new prehospital CPAP equipment will be required in five years) and sharing the overall costs out amongst the number of patients that would benefit from the service over this time period.

Training costs for initial and new staff to use prehospital CPAP were provided by expert opinion from WMAS. The estimate of £70,484 was again lower than the previous economic model, but was informed by the training model used in the ACUTE pilot trial. This assumed an initial 0.5 hour training session, with one further refresher session during the 5 year technology lifespan.

Prehospital CPAP device costs were dependent on the number of ambulances that would need to be equipped with CPAP devices, the incidence of ARF, and the cost of disposable CPAP devices. An estimate of 550 ambulance was provided by WMAS. A CPAP device cost of £17.25 was quoted from SP Services, the UK suppliers of the O-Two unit. This compared to a £513.49 unit cost for the Boussignac CPAP system used in the previous model. The incidence of ARF patients who will benefit from prehospital CPAP is one of the key parameters in the model, as the unit cost of prehospital CPAP is estimated by dividing the total costs of a prehospital CPAP to the ambulance service by the number of CPAP devices used. The mean incidence rate reported in the ACUTE study was 18.2 per 100,000 people per year (95% CI 17.1 to 19.3 per 100,000 people per year). Uncertainty in the cost of delivering prehospital CPAP per patient was estimated by changing the cost of device by £5 in either direction, and by using a range of incidences (17.1 to 19.3 per 100,000) based on the 95% confidence limits of the incidence estimate.

This resulted in a final CPAP cost per patient ranging from £26.53 to £39.57. This was assumed to be normally distributed around the mean of £33.00 with a standard deviation of £3.30. The resulting costs were much lower than in the previous analysis because a newer CPAP device was used (O-Two v Boussignac CPAP system). This was much cheaper and required less training, so that the reduced cost was roughly half attributable to reduced device cost and half to reduced training costs.

Table A1: Breakdown of prehospital CPAP costs

| **Breakdown of device costs** | | | | | |
| --- | --- | --- | --- | --- | --- |
|  | Number of devices | Source | Unit cost | Source | Total Cost |
| Cost of Prehospital CPAP device | Number of ambulances (550) that need adding the CPAP device | Expert advisory input -WMAS | £17.25 | O-Two/SP Services^14^ | 550*£17.25 |
|  | usage over five years = 5*1017 (where 1017 is number of patients per year) | Expert advisory input | £17.25 | O-Two/SP Services^14^ | £17.25*5*1017 |
| Total cost of the device | | | | | £97,204 |
| Staff costs | | | | | |
|  | Resource usage | Source | Staff cost (per hour) | Source | Total Cost |
| Initial Training costs | 0.5 hours training  Paramedics n=1384  Technicians n=1002  Student Technicians n=816 | Expert advisory input -WMAS | Band 6 - £21.76  Band 5 - £17.42  Band 4 - £14.40 | Expert advisory input | (0.5*£21.76*1384) +  (0.5*£17.42*1002)  +  (0.5*£14.40*816)  = £29,660 |
| Costs of ongoing training for new staff | 0.5 hours training  Technicians n=90 per year  Student Technicians n=280 per year | Expert advisory input -WMAS | Band 5 - £17.42  Band 4 - £14.40 | Expert advisory input | (0.5*£17.42*5*90)  +  (0.5*£14.40*5*280)  = £14,129 |
| Refresher training for existing staff (using 10% attrition rate to account for promotion, retirement, leavers etc) | 0.5 hours training  Paramedics n=1245  Technicians n=902  Student Technicians n=735 | Expert advisory input -WMAS | Band 6 - £21.76  Band 5 - £17.42  Band 4 - £14.40 | Expert advisory input | (0.5*£21.76*1245) +  (0.5*£17.42*902)  +  (0.5*£14.40*735)  = £26,695 |
| Total staff costs | | | | | £70,484 |
| Total costs of prehospital CPAP | | | | | £167,688 |
| Number of patients in five years (5 years*1017 per year) | | | | | 5085 |
| **Cost of prehospital CPAP per patient** | | | | | £33 |
