# Supplementary material for: Cost-effectiveness of out-of-hospital continuous positive airway pressure for acute respiratory failure: decision analytic modelling using data from a feasibility trial
Source: BMC Emerg Med. 2021 Jan 25;21:13. doi: 10.1186/s12873-021-00404-8 (PMC7836588; doi:10.1186/s12873-021-00404-8)
Supplement: Supplementary file 2 — Additional file 2: Appendix 2. Value of Information analyses. Provides more detail about the methods and results of value of information analyses. [file 12873_2021_404_MOESM2_ESM.docx]

## Appendix 2: Value of Information analyses

### Expected value of perfect information

Reflecting the uncertainty in effectiveness of prehospital CPAP, together with the large potential opportunity losses from making the incorrect adoption decision, base case individual EVPI was considerable at the NICE willingness to pay threshold: £300 at λ=£20,000. Given the relatively large annual population with ARF eligible for prehospital CPAP treatment (11,000 across England and Wales), and long time period over which the technology is likely to be applicable (5 years), base case population EVPI was also correspondingly large in the base case analysis: £16.5 million at λ=£20,000/QALY. This indicated it would be worth spending up to £16.5 million on research investigating the effectiveness of prehospital CPAP in ARF. Population EVPI was substantial at thresholds under £8,000, but decreased to a minimum at a threshold of £10,000/QALY, where there was little uncertainty about whether to adopt or reject CPAP based on existing evidence. Population EVPI continued to increase at higher cost-effectiveness thresholds, representing rising uncertainty. Base case population EVPI is presented in Figure A1.


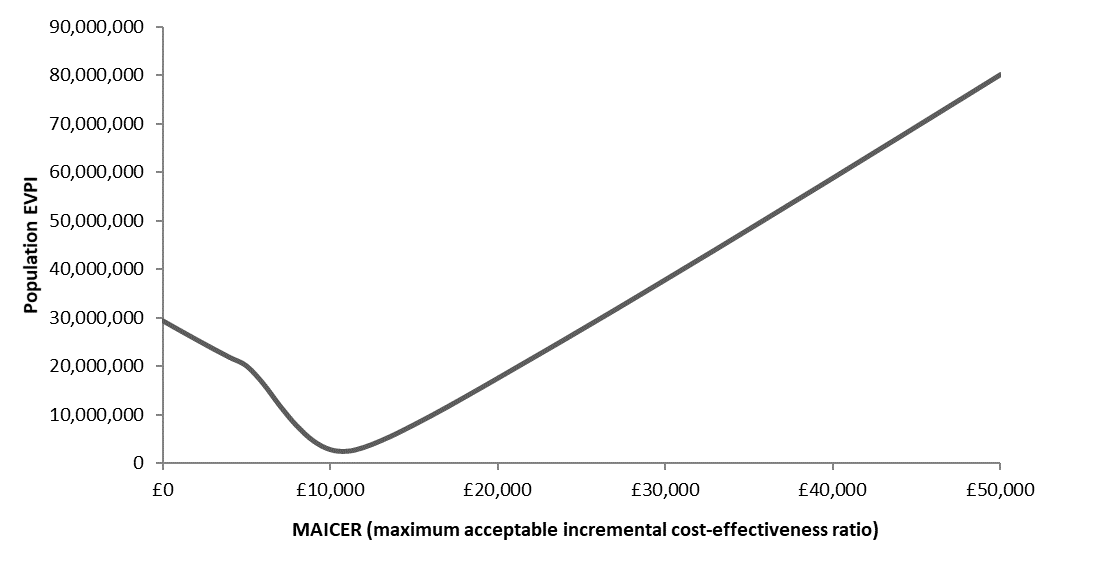


Figure A1. Base case population EVPI

For the updated network meta-analysis scenario analysis individual EVPI was also appreciable at λ=£20,000: £67.6; corresponding to a population EVPI of £3.72 million. Population EVPI for this scenario analysis are presented in Figure A2.


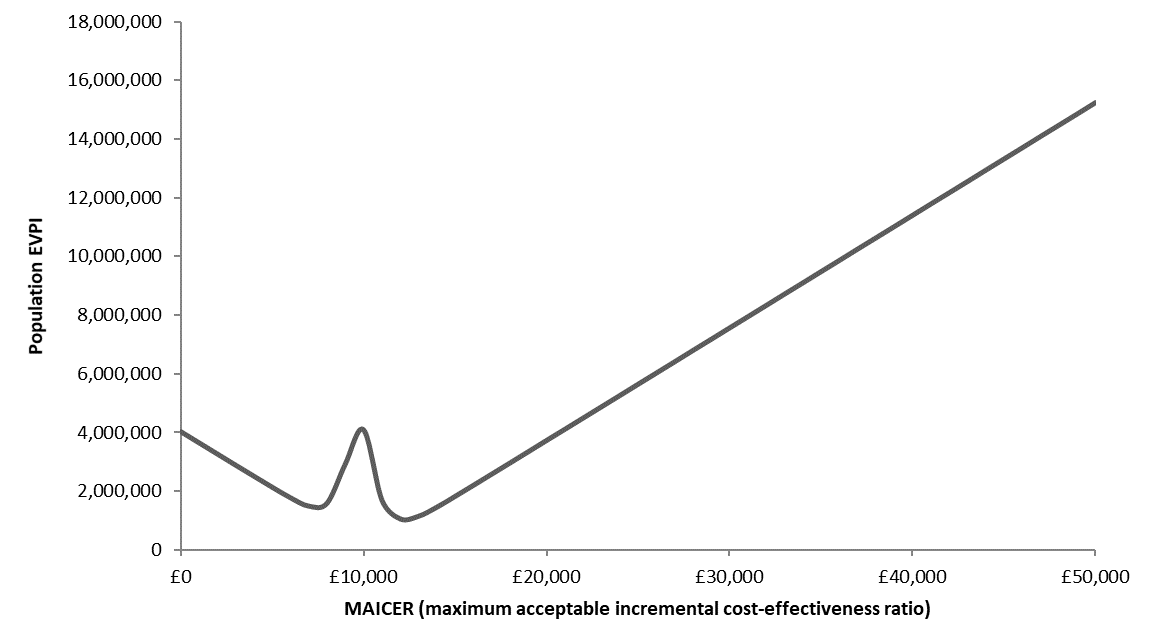


Figure A2. Updated network meta-analysis scenario population EVPI

### Expected value of partial perfect information

The population EVPI places an upper limit on the total value of addition research relating to a specific decision problem, but does not indicate where future research may be beneficial. The population expected value of partial perfect information (EVPPI) indicate the value of reducing the uncertainty surrounding particular input parameters in the decision model. Ten parameters, reflecting targets for potential future research designs, were considered in the EVPPI analyses: Baseline mortality and risks; relative effectiveness for mortality and intubation; costs of prehospital CPAP, hospitalisation, intubation and long term survival; life expectancy; and lifetime quality of life.

The expected value of partial perfect information (EVPPI) associated with each of these parameters in the base case are illustrated in Figure A3. At the threshold of £20,000/QALY, individual EVPPI associated with effectiveness of CPAP in reducing mortality was £299.6 and individual EVPPIs for all the other parameters were zero. The population EVPPI for CPAP effectiveness on mortality in base case was £16.5 million.

Figure A3. Base case population EVPPI at a £20,000 per QALY cost-effectiveness threshold

The expected value of partial perfect information (EVPPI) for model parameters in the updated meta-analysis scenario analysis are illustrated in Figure A4. At the threshold of £20,000/QALY, individual EVPPI associated with effectiveness of CPAP in reducing mortality was £67.6, and EVPPIs for all the other parameters were zero. The population EVPPI for CPAP effectiveness on mortality in this scenario analysis was £3.72 million.

Figure A4. Updated network meta-analysis scenario EVPPI at a £20,000 per QALY cost-effectiveness threshold
